# Perceptions and experiences of community first responders on their role and relationships: qualitative interview study

**DOI:** 10.1186/s13049-018-0482-5

**Published:** 2018-02-05

**Authors:** Viet-Hai Phung, Ian Trueman, Fiona Togher, Roderick Ørner, Aloysius Niroshan Siriwardena

**Affiliations:** 1 Community and Health Research Unit (CaHRU), School of Health & Social Care, University of Lincoln, Lincoln, LN5 7AY UK; 2 School of Health & Social Care, University of Lincoln, Lincoln, LN5 7AY UK

**Keywords:** First responders, Prehospital care, Urgent care, Ambulance care, volunteers

## Abstract

**Background:**

Community First Responders (CFRs) are lay volunteers who respond to medical emergencies. We aimed to explore perceptions and experiences of CFRs in one scheme about their role.

**Methods:**

We conducted semi-structured interviews with a purposive sample of CFRs during June and July 2016 in a predominantly rural UK county. Interviews were transcribed verbatim and analysed using the Framework method, supported by NVivo 10.

**Results:**

We interviewed four female and 12 male adult CFRs aged 18–65+ years with different levels of expertise and tenures. Five main themes were identified: motivation and ongoing commitment; learning to be a CFR; the reality of being a CFR; relationships with statutory ambulance services and the public; and the way forward for CFRs and the scheme. Participants became CFRs mainly for altruistic reasons, to help others and put something back into their community, which contributed to personal satisfaction and helped maintain their involvement over time. CFRs valued scenario-based training and while some were keen to access additional training to enable them to attend a greater variety of incidents, others stressed the importance of maintaining existing abilities and improving their communication skills. They were often first on scene, which they recognised could take an emotional toll but for which they found informal support mechanisms helpful. Participants felt a lack of public recognition and sometimes were undervalued by ambulance staff, which they thought arose from a lack of clarity over their purpose and responsibilities. Although CFRs perceived their role to be changing, some were fearful of extending the scope of their responsibilities. They welcomed support for volunteers, greater publicity and help with fundraising to enable schemes to remain charities, while complementing the role of ambulance services.

**Discussion:**

CFR schemes should consider the varying training, development and support needs of staff. CFRs wanted schemes to be complementary but distinct from ambulance services. Further information on outcomes and costs of the CFR contribution to prehospital care is needed.

**Conclusion:**

Our findings provide insight into the experiences of CFRs, which can inform how the role might be better supported. Because CFR schemes are voluntary and serve defined localities, decisions about levels of training, priority areas and targets should be locally driven. Further research is required on the effectiveness, outcomes, and costs of CFR schemes and a wider understanding of stakeholder perceptions of CFR and CFR schemes is also needed.

## Background

A Community First Responder (CFR) “*is a member of the public who receives basic emergency care training and volunteers to help their community by responding to appropriate medical emergencies while an ambulance is en route*” [1]. CFRs were introduced to help improve response to emergencies such as out-of-hospital cardiac arrests and more recently to help ambulance services reduce response times to incidents more generally, especially in rural areas [2, 3].

There is evidence that in rural communities in particular, having CFRs can reassure patients [4, 5] and reduce time to defibrillation [3]. Intervening in the *chain of survival* by reducing the time to defibrillation improves survival rates [2, 6, 7]. CFRs primarily complement the work of the ambulance service by stabilising patients’ conditions, performing basic clinical procedures and recording vital signs before handing care to paramedics [4, 8]. Their role has expanded over time and in some geographical areas to encompass other conditions [4, 5].

CFR schemes have been supporting prehospital emergency care since the 1990s [9]. In 1999, the United Kingdom (UK) government encouraged ambulance services to make use of CFRs, especially in rural locations, to help them meet the 8 minute response time targets for emergencies [4, 5]. There is evidence that CFRs are used widely across most ambulance services in the UK [4 5]. In early 2014, there were 2431 CFR schemes, using over 12,000 volunteers in the UK [8], although data suggest that they still only respond to a small proportion of all calls [1]. Each scheme serves a particular defined geographical area with particular needs and priorities, which shape their operational procedures, training and support to volunteers [1]. CFRs attend to incidents in their own time, using their own vehicles and selecting their hours of duty by notifying when they are available [5].

The National Association of Community First Responders (NACFR), which was set up to develop national standards for CFRs across the UK, became a member of the National Council of Voluntary Organisations (NCVO) in November 2014 [10] and from February 2015, was recognised as a charity [11]. CFR schemes typically receive their funding from fundraising and public sources, the balance of which varies between schemes [4, 12, 13]. 

Some UK regions, such as the East Midlands, have both independent CFR schemes, such as LIVES, and schemes run by ambulance services. LIVES works with, but has autonomy from, the regional ambulance service, East Midlands Ambulance Service NHS Trust (EMAS). Their autonomous status means that the Care Quality Commission (CQC) effectively considers CFR schemes to be private ambulance providers. Consequently, the CQC quality indicators do not fully capture the nature of what CFR schemes do [14].

A recent scoping review [15] established an evidence base on CFRs and schemes in the UK. We aimed to build upon this by exploring the perceptions and experiences of CFRs in one scheme.

## Methods

### Study design and setting

We conducted a qualitative interview study with a purposive sample of CFRs from a single scheme - LIVES - in Lincolnshire, UK.

Semi-structured interviews took place at CFR scheme and university premises during June and July 2016. Two researchers (FT and VHP), neither of whom had any prior relationship with the interviewees, undertook separate face-to-face individual interviews.

The scheme has over 700 volunteers who attend around 20,000 calls annually [16]. There are three levels of expertise within this CFR scheme from Level 2 CFRs who provide basic patient assessment, resuscitation, defibrillation, first aid and oxygen therapy to Level 3 CFRs who are able to manage trauma and attend paediatric incidents, progressing to Level 4 CFRs who have had extra training in motor vehicle entrapment and extrication as well as administration of specific pain relieving drugs.

### Participants

We interviewed participants of different ages, sex, length of experience, and level of expertise. Twenty three CFRs initially expressed an interest in taking part in the study from which 16 agreed to take part and were interviewed.

### Data collection and analysis

Each interview was semi-structured, lasting 30–90 min, using an interview schedule to guide, but not dictate, the discussion. Interviews were subsequently transcribed verbatim and coded thematically in NVivo 10 [17] using framework analysis [18]. Two researchers (VHP and IT) coded the transcripts between them. These transcripts were then analysed by VHP, IT, NS and RO. The resultant themes were discussed and agreed by all four researchers. FT and VHP, who conducted the interviews, are experienced in conducting qualitative interviews. NS, FT and VHP are experienced in analysing qualitative data. IT is a trained CFR within LIVES, while RO is a consultant psychologist, whose expertise was valuable during the analysis.

## Results

We interviewed four female and 12 male CFRs, aged ranging from 18 to 24 years up to 65 years and above, with varying expertise and length of experience from 3 months to 16 years (Table 1). Ten out of the 16 interviewees were aged 55–64 years or over, while 13 of the interviewees were at either Level 3 or Level 4.

**Table 1:** Participant characteristics

Category	Frequency (per cent)
Sex	
Male	12 (75)
Female	4 (25)
Age range	
18–24 years	1 (6)
25–34 years	1 (6)
35–44 years	1 (6)
45–54 years	3 (19)
55–64 years	7 (44)
65–74 years	3 (19)
Level of expertise	
Level 2	3 (19)
Level 3	9 (56)
Level 4	4 (25)

The main themes from the interviews were organised as follows: motivation and ongoing commitment; learning to be a CFR; the reality of being a CFR; relationships with statutory ambulance services and the public; and the way forward for CFRs and the scheme.

### Motivation and ongoing commitment

Interviewees described how and why they decided to become a CFR as well as their reasons for remaining so.

#### Paths to volunteering

Volunteers usually found out about the CFR scheme through talking to existing CFRs or seeing publicity material. They followed up their initial interest by making further enquiries with existing CFRs and attended meetings that explained what the service did. Thereafter, volunteers acquired the skills needed to become a CFR by undertaking the requisite training.

*When I realised it was a community first responding scheme, I became a little bit interested. I spoke to my two friends about it, who strongly recommended it and invited me to a meeting. I came along to one of the meetings and spoke to a few members. They explained what they did and how things work and my interest piqued really. I said I was available and joined up*. Male, 18-24 years.

#### The value of being a CFR

The primary motivation for becoming and remaining a CFR was altruistic, but volunteers also derived personal satisfaction and self-esteem from helping others. Some participants also saw the role as a route towards a future career as a health professional.

*The best bit about it, and it really is the best bit, is when you realise you’ve made a difference, you’ve done something good*. Male, 55-64 years.

*We’ve got a student nurse and a lad who is soon to start who works in an ambulance service, he’s a student paramedic, and myself – we’re all going into the healthcare profession at some point*. Male, 18-24 years.

A key motivation was to make a difference to patients. While CFRs received anecdotal feedback from family or friends of patients that they had attended, the absence of formal feedback mechanisms meant that there was no means of recognising their contribution, which participants agreed should be addressed.

*To be honest with you, because you’re in the local village, you may bump into people that you’ve treated – which any doctor does or anything – and it’s patient confidentiality, that’s fine. And they will inevitably say, “Thank you very much.” But we don’t get any phone calls or anything like that, as a general rule*. Male, 45-54 years.

*No, but I think you can ask for it [feedback], but you’d only get it if they’d offered it to head office, because it’s all data protection*. Female, 55-64 years.

#### Balancing volunteering with personal life

Work as a CFR was accommodated flexibly within participants’ other work commitments or caring responsibilities. These varied greatly. CFRs were attracted by this flexibility, but once they made themselves available, they had to be ready at short notice. As with any emergency service, the number of calls was irregular, which volunteers accepted as inevitable.

*So, if I know for example that I’ve got six hours free, then I can log on for that six hours, and when I need to go, I just tell them that I need to go and that’s the end of it. There’s no rota; there’s no set period of time that you have to be logged on for, so it fits perfectly. It’s basically I can do it when I want*. Male, 18-24 years.

### Learning to be a CFR

Once they decided to become a CFR, participants had to undertake a period of initial training and assessment. The level of training they undertook determined how skilled they would be and consequently, the types of incidents they would be able to attend. Beyond basic skills training, CFRs developed further expertise through a mixture of local group training events and study days.

#### Training preferences

CFRs particularly valued what they felt to be realistic simulations of the types of incidents they would eventually attend which enhanced their preparedness for attending real-life situations. Some respondents recognised that simulations did not necessarily prepare volunteers for the full range of incidents to which a CFR could be called but accepted that it was unrealistic to expect scenarios to cover all eventualities.

*Very real, because they are highly experienced training officers that see those incidents every single day, so they know how to play role (sic) it, if you know what I mean. And a lot of the… I don’t know what you’d call it. They’ve got things up at head office: like there’s a smashed-up vehicle*. Male, 25-34 years.

*You don’t know what you’re going to get when you log on duty, but in real life – especially in my area, because it’s very rural, it’s very quiet, you don’t get many assaults or drug abuse. They will be more age related problems, so what you might get on a scenario day isn’t necessarily a true reflection of real life where you live*. Male, 65-74 years.

While they valued the realism of scenario-based training, participants spoke also of needing other practical training that dealt with specific skills that their role required, such as handing over care to ambulance staff.

*Handover-wise, I think if we were trained to give handovers that would be very, very useful, because we are not actually training on how to do a handover. So we don’t know the order that the paramedics want the statistics or anything like that. We don’t actually get told how to do that; it is something that you learn*. Male, 55-64 years.

#### Progressing as a CFR

Some participants welcomed the opportunity to progress through CFR skill levels by undertaking additional training to enable them to attend to, and deal confidently with, a wider range of incidents. The scheme encouraged respondents to progress according to their desire to advance: some wished to progress more quickly, feeling constrained by rules governing progression while recognising that this was necessary.

*Level 4 is a lot more advanced. Again, it’s three days of actual training but you have to go through a far, far higher level of diagnostic training. You also have to be able to give certain drugs. They’re able to give Entonox, which is a pain relieving gas – the old gas and air. They can also give salbutamol for asthma as well as glucagon for someone who is hypoglycaemic…* Male, 45-54 years.

*Most of the guiding lights, if you like, of LIVES have been clinical professionals, massively experienced in their fields, massively competent, magnificent at their job as clinical professionals. But their mind-set, if you like, is onwards and upwards. If you’re Level 2, you must be striving to Level 3 and Level 3 should be striving to Level 4, constantly getting more skills, more knowledge and progressing onwards and upwards*. Female, 55-64 years.

Participants recognised the importance of maintaining existing as well as learning new skills. There was a perceived risk that constantly upskilling would eventually blur the distinction between CFRs and ambulance service staff which CFRs were keen to preserve.

…*some members of staff think it is a cheap ambulance service and some don’t agree with the principle*. Male, 25-34 years.

*The training needs to be more about keeping them competent in the basics so that they can build on if they want to, rather than always give them extra to build on, build on, build on*. Male, 55-64 years.

#### Suggestions for improving training

Participants generally rated the training and the trainers very highly. They valued the scenario-based training as well as training on particular practical skills but were also concerned about the lack of interpersonal skills training. CFRs felt their organisation assumed they had the requisite communication skills whereas they perceived a need for both communication and clinical skills training.

*I think they are under the impression that it’s something that you either have or don’t have and that’s where it ends. They very much focus on the clinical side of things. I think even just half a day with patient communication would be beneficial.* Male, 65-74 years.

*Some training on how to deal with, not irate but upset family members or concerned family members would be something that I would be interested in even if it was just something to touch on for an hour or so in a training day.* Male, 45-54 years.

### The reality of being a CFR

After becoming CFRs, the next step for volunteers was to attend incidents, some of which were emotionally distressing.

#### Being first on-scene and alone

The nature of the CFR role meant that participants were often first on-scene and alone. This placed great responsibility on them, which in turn, heightened their stress and anxiety. The CFRs described their experience of being the first person on scene, with their primary responsibility being to stabilise the patient’s condition before ambulance service crew arrived.

*I’m the first one on scene then I’ll be doing what I can for the patient and when they come in the door behind me, it’s my job, it’s my responsibility to the patient, to them and to LIVES, if you like, to give a handover to the ambulance service and say, “This is what I’ve got. This is what I found when I got here. This is what I’ve been doing since and this is the state they’re in now*.” Male, 55-64 years.

#### Preparing for and attending incidents

The scenario-based training, which aimed to be as realistic as possible, was welcomed by participants, but the wide range of incidents attended, were not always covered. To prepare themselves, volunteers kept an open mind about what they would be called to. This was especially necessary since information from control rooms sometimes differed markedly from the actual incident.

*It’s not something you can prepare for emotionally, I don’t think, because you don’t know what you’re going to come across – is the best I can answer it. You don’t know what you’re going to see, you don’t know what you’re going to have to do, so to try and prepare emotionally for it would actually have a detrimental effect*. Male, 18-24 years.

#### Emotional support

Some incidents could exact a high emotional toll, and CFRs could access a range of support mechanisms to help them deal with these experiences. The CFRs valued the flexibility and informality of these support mechanisms.

*As I have said from the outset, there are numerous members of staff at HQ who have all said, “This is our phone number. I don’t care what time of day it is; if you have a call and you need to talk about it, ring me and I’ll answer the phone*.” Female, 55-64 years.

#### Fundraising

While the CFR scheme received some statutory funding, they were required to augment this through fundraising activities [4, 13, 14], an aspect of the role which participants did not enjoy but recognised the need for it to ensure effective functioning of the organisation.

*Because you’ve got to add fundraising on to top up the LIVES fees, so the commitment is quite a lot*. Male, 45-54 years.

### Relationships with statutory ambulance services and the public

There were a number of issues around the relationship between CFRs and ambulance staff as well as the nature of their relationship with the patients they served. These centred on the lack of public recognition, the inability to distinguish between CFRs and ambulance staff and blurred lines of accountability, which sometimes caused conflict between CFRs and the ambulance service.

#### When CFRs and ambulance staff complement each other

CFRs serving a defined area responded to calls from the ambulance control room when there were incidents in that area. A timely response by CFRs helped stabilise a patient’s condition, provided essential information and helped the ambulance service meet response time targets for emergencies, easing the pressure on stretched ambulance resources.

*You know, you give them as much statistical information as you can. So it’s: pulse rate, respiratory rate – all that kind of information. Then they know, straight away, a lot of vital information for their assessment, so that they haven’t got to do the tests immediately. If they have certain information, they know what route to go down probably quicker than if they’d just turned up and have to start doing everything themselves*. Female, 55-64 years.

An effective working relationship between CFRs and ambulance staff also depended on staff attitudes towards each other. The relationship worked well when CFRs were keen to learn from ambulance service staff and the latter were keen to pass on their skills to the former.

*It’s quite nice actually. It makes a change because if you register a tiny bit of interest, they will explain everything. It’s a good learning curve*. Male, 18-24 years.

*Sometimes other crews will say why don’t you stay and you can learn this or you can help us do that and actually involve the responder and that actually builds up the responders confidence up and they think 'Oh' I actually helped a bit extra and I have done something above my skills set I’ve been supported by a paramedic or they might say if it’s a poorly patient can you travel to hospital with us in case we need your help on the way and actually it builds up the CFR and they think I was asked to go to hospital and that was really good and they felt confident in me asking to go and support them on a job*. Male*, 25*-34 years.

An effective relationship between the two also relied on mutual trust. CFRs had to know when to hand over to ambulance service staff who were better equipped to deal with the situation. Ambulance service staff had to trust that the CFRs had done what they needed to do correctly to alleviate the patient’s situation, ready for them to take over on arrival.

*I’ve always regarded myself as just being the trained amateur and once they come in the door, I’ll tell them what I’ve found, I’ll tell them what I’ve done, I’ll tell them what I’ve got and then I’ll defer to them straightaway because they’re professionals, they've got the registration, they’ve got all the practice and all the kit. At that point, I just become whatever they want me to be*. Male, 55-64 years.

#### Conflict between CFRs and the ambulance staff

The relationship between CFRs and the ambulance service sometimes did not work effectively. A breakdown in communication between the CFR, the control room and ambulance service staff sometimes led to CFRs being stood down from a job. Whilst most volunteers were happy with this arrangement, others expressed frustration at being stood down after they arrived at the address with the ambulance already there. Some participants recognised that, at times, they may be called out of their area to assist when the ambulance service had been stretched. Other respondents were frustrated that they remained on call and were not being used. This suggested a feeling that they were not trusted enough.

*The very, very first call I had, I got there, the ambulance was already there – and when an ambulance is there you make contact with the crew, basically, “Do you need any help?” Nine times out of ten they’ll say, “No, we’re fine,” occasionally, “Yes please, come in.” On this occasion, “No, we don’t need any help,” so I turn around and I drove home and I couldn’t get out of the car, I was absolutely shaking*. Male, 65-74 years.

Lack of trust could also undermine the relationship between CFRs and ambulance staff. The different skill levels sometimes had the unintended consequence of reinforcing the hierarchy between the two. This also manifested itself at a macro-level with the failure of the ambulance service to fully acknowledge the role that CFRs played in meeting response time targets. Whether the ambulance service did this consciously or not, the failure to acknowledge the CFR contribution to meeting response time targets, allied with the low public awareness of what they do, was perceived to undervalue their work and hinder recruitment.

*I think what would help us is if the ambulance service acknowledged us a little bit more. When they do their press conferences saying, “We met this statistic and that statistic,” I think that would help a big deal, because if we are acknowledged by them… However, I think that is in the pipework and I think it is something that won’t happen overnight.* Male, 18-24 years.

#### Relationship between CFRs and patients

Because the CFR scheme was often based in small rural communities, participants were often well known to patients. CFRs within such communities provide reassurance that someone among them has the skills to help people out of a distressing situation. In some cases, CFRs attended to people they knew. This potentially posed emotional challenges for the CFRs. While they maintained an open mind, as they did in all incidents they attended, the impact of such incidents on them was different.

*Once the treatment from you has stopped personally, then you start reflecting on it and thinking “Hang on a sec, they’re my friend. Is there anything that I could have done differently?” But the treatment level between a friend and somebody that you have never met before does not change. It doesn’t matter that it’s a friend*. Male, 55-64 years.

#### Lack of public recognition of CFRs

Patients often expected a paramedic to arrive first, which reflected the low public recognition and awareness of CFRs. In some instances, patients were disappointed with the response they received, which CFRs felt undervalued their work. This is where communication skills might help CFRs explain their purpose to tackle these preconceptions. Sometimes, the distinction between CFRs and the ambulance service did not matter: whether they worked for the CFR scheme or the ambulance service. In those instances, the most important thing for patients was to have someone on hand with the skills to help them [22].

*Well I tend to wear my green polo shirt and black trousers or something similar. Roughly, they dial 999 and five minutes later, I turn up in my car and walk through the door. They often say “the doctor’s here” or “the paramedic’s here” and I reply, “no, I’m a LIVES responder”. But they don’t care at that point. There’s somebody here. They don’t care who it is*. Male, 45-54 years.

### The way forward for CFRs and the scheme

Having discussed their motivations to become CFRs, as well as their experiences, in particular, their relationship with the ambulance service and patients, interviewees were then asked to envisage how the CFR role and the scheme could evolve.

#### The evolving role of the CFR

Participants were keen for their scheme to be clear about what they wanted from their CFRs. The pace of change had been so rapid that some interviewees expressed concern about the impact on their role and identity. CFRs feared that the role may extend beyond its current remit, focusing on less urgent calls in place of an emergency ambulance response.

*First off decide what LIVES is about. It started off as doctors and medics, healthcare professionals using their skills in their off duty time. Then it became community first responders as well. The community first responders idea keeps on changing and varying. That, I think, is where, if there’s a problem, that’s probably where it lies, is what do we want from the community first responders? What are we actually asking them to be able to do?* Female, 55-64 years.

*It’s a tricky one because for us to improve any further, we would be taking it away from the realms of it being in your spare time as a community first responder. I think if we were trying to improve or trying to do any more, we would start having to change the qualification levels. It would start becoming far too formal. I think it would be too close to becoming an ambulance service than a community responding charity.* Male, 18-24 years.

#### Complementary but distinct

Some participants spoke of their concerns that they did not want their scheme, a charity, to become more closely aligned with the ambulance service. This was linked to fears about the pace of change and the scope of the organisation. Volunteers wished to be seen as complementary rather than as an extension of the statutory service.

*I’m from the South West. The RNLI is down there, everybody knows about the sea and the Lifeboat Institution is revered. They’re all volunteers, they’re all charity funded, they do a wonderful thing. But they’re the only people in the field doing it. There is no statutory provided organisation which they work around. LIVES is like the RNLI. Ours should be a bonus to the system, not a fundamental part of it.* Male, 45-54 years.

It was felt that changes needed the consent of volunteers and that key towards achieving this was better communication with headquarters. Some felt that communication could be improved.

*I think we could do with more information. Sometimes the two-way bit is hard to fathom. There’s also this feeling, sometimes, that people forget that the responders are all volunteers. I suppose, in a sense, that’s a bit of a backhanded compliment because it means they think of them as professionals. Male, 55-64 years.*

Volunteer support [subheading] [next line] Participants expressed the need for greater support, for example through the role of First Responder Development Officers (FRDO).

*I think for the FRDOs who have so many groups as well, they are too far out, they’ve got too many groups to do. We need somebody a little bit more localised, it’s that stepping stone between us and head office. And a more localised holding point for equipment, so that we’re not travelling 45 minutes an hour to pick up essential stuff. We keep a little bit, we do keep a little bit in our own homes, but I’m talking about oxygen – stuff like that.* Female, 55-64 years.

#### Publicising the work of the CFR scheme

Participants were frustrated that the work of their scheme was often not recognised by the local population, fearing this might have a negative impact on recruitment. Some felt that, in the public mind, CFR scheme and ambulance service were one and the same, with patients often assuming that the CFR was from the ambulance service or a member of ambulance staff. To rectify this their scheme was raising public awareness of its identity and purpose, through more effective marketing. This was welcomed, but interviewees felt more needed to be done.

*The two main things I would like to see improved are the awareness and recruitment*. Male, 45-54 years.

*I think probably most people only know about LIVES if you’ve had a LIVES responder out to your friends or family, or if, you know, you know somebody personally. But, I mean, a lot of people don’t really know what LIVES is or what LIVES means. Do you know what LIVES stands for?* Female, 55-64 years.

#### Future funding arrangements

Participants were hopeful that fundraising would be improved by having a dedicated fundraising officer rather than just CFRs themselves.

*Redeveloping the brand and merchandise and just marketing the whole… you know, getting the message out to everybody. And the way it’s… the fundraising: that’s all going to be changed because, at the moment, we all do our own fundraising for our own group*. Female, 55-64 years.

Most participants felt that the CFR scheme should be viewed as a provider separate from the ambulance service and a charity in the same way as the air ambulance was. CFR schemes had to raise substantial funds themselves and the responsibility for this lay with individual CFRs as well as the CFR scheme which had raised its profile by having a bigger presence at fundraising local events.

*And so if people view us as part of the ambulance service, then they view us as centrally funded and therefore, we don’t, in their eyes, don’t look the same as the air ambulance. Everybody knows it’s not centrally funded and needs lots of money to keep the gents in the air*. Male, 45-54 years.

*I’ve got one coming up this Saturday, there’s a drive sale where they raise thousands and thousands of pounds for air ambulance, not a single person would have put in for LIVES. But this year I’ve got a table*. Female, 55-64 years.

## Discussion

### Main findings

CFRs in this study felt they gave something back to their local community by helping others in times of acute need [19–22]. Previous studies found that volunteers became CFRs primarily for altruistic reasons, such as “*community spiritedness*” [23], wishing “*to give something back to the community*” [19], and “*the desire to help*” [24] others, e.g. after witnessing a neighbour who died “*because nobody could attend to him quickly enough*” [24]. While altruistic motivations were important, volunteers also derived individual satisfaction, for example, “*enjoyment of contact with people*” [23]. The flexibility of the role, to fit in with “*other aspects of members’ lives*” attracted volunteers, both to become and remain a CFR [24]. Some, particularly younger, participants also saw experience as a CFR along the pathway to a career in the health professions.

CFRs felt that training using simulations of incidents was sufficiently realistic to prepare them for attending real-life emergencies. While they welcomed the practical value of the training given, the CFRs felt that communication skills were a significant omission from current provision. While the interviewees in this study supported the training they received, in other schemes, some volunteers, while they shared their desire for more training to progress, “*had not received ongoing training, leaving them feeling unsupported*” [20]. Moreover, irregular provision of ongoing training, for particular skills, such as using Automated External Defibrillators (AEDs) increased the risk of their skills becoming obsolete. This consequently could limit their ability to attend incidents because “*many stated that they would hesitate to use an AED in a different location in case they were not ‘allowed’ to do so*” [25]. There were real fears about potential litigation if their AED training was no longer recognised because of the lack of refresher training [25].

By arriving first on-scene and treating patients before the ambulance service arrives, CFRs may improve patients’ outcomes, especially in relation to cardiac arrests [7, 21]. However, it is difficult to definitively say what the impact on patient outcomes has been because limited data exist to link prehospital incidents with what subsequently happens to patients [5]. While CFRs felt they contributed to improved patient outcomes, the need for formal feedback mechanisms on the effect of their attendance is reflected in the wider literature [19, 20].

While some CFRs were keen to progress quickly through the different levels [5], others were also keen to maintain their existing skills. Continual upskilling of volunteers risked making them indistinguishable from ambulance service staff. This went against CFRs’ desire to complement but remain distinct from the ambulance service.

CFRs preferred having more flexible peer support to help them deal with emotionally draining incidents. They adopted a very pragmatic approach and kept an open mind when responding to incidents. This made them seem resilient and prepared for anything they were likely to come across, regardless of what the control room had told them.

Participants carried out fundraising and marketing at local events to raise the perceived low public profile of the CFR scheme. Previous research also suggests a need for greater recognition and public understanding of CFRs and CFR schemes, which could help to bridge the disconnect between the CFR scheme and their community [20]. That said, some CFRs felt that patients felt reassured that they had someone on hand with the skills to help them, despite not being a professional health worker [5, 22]. CFRs sometimes felt undervalued by the ambulance service, who they felt perceived them to be a cheaper, less skilled workforce. An effective explanation of the purpose of CFRs could also help to minimise ambulance staff suspicion that the volunteers were encroaching on their territory and help foster a better working relationship between the two organisations [19].

### Implications for future policy, research and practice

CFR schemes need to accommodate the differing needs of CFRs to develop further skills. This requires a clear vision of what they expect CFRs to do, and in turn, requires them to identify their skill needs, which might vary between areas with different needs. CFR schemes also need to provide communication skills training alongside clinical training which would enhance volunteers’ ability to communicate effectively with patients and ambulance staff [26]. While the interviewees in our study valued having flexible support mechanisms to deal with particularly distressing incidents, CFR schemes may need to consider how to identify stress and provide emotional support to volunteers [27].

CFRs in our study wanted their organisation to be complementary to ambulance services while remaining distinct from it; they felt ambulance services did not want volunteers to encroach on their territory. CFRs are separate from ambulance services, but are bound by regulatory CQC quality indicators similar to those that measure the performance of ambulance services. Clarifying the lines of accountability would help to minimise potential tensions between CFRs and ambulance staff.

There is a lack of data on the contribution of CFR actions to outcomes, which makes it difficult to know what their effect is [5]. Existing data on numbers of calls attended to by CFRs could be supplemented by data linking prehospital care provided by CFRs to patient outcomes. Better data linkage could measure the contribution of CFRs to meeting response time targets and help assess cost-effectiveness of CFR schemes. CFRs felt they would benefit from formal feedback mechanisms, and schemes would need to consider how these could be implemented.

Any future expansion of CFR schemes needs to explore how CFR schemes currently operate, what constitutes best practice and how future schemes could be organised and develop. Further evidence is needed on effectiveness of CFR schemes, particularly in relation to response times, survival and the experience of service users, while the views of ambulance services and commissioners are also important.

### Strengths and limitations

The multi-disciplinary nature of the research team brought different perspectives to the analysis. In doing so, it enriched the study. This was a study of one rural CFR scheme. While there maybe commonalities between schemes, as shown by similarities between findings from this interview study and a recent scoping review, given their local nature, we must be cautious about the extent to which the findings can be extrapolated more widely. CFR schemes are area-specific, which will influence their priorities. In this context, it is important for each scheme to be clear about the purpose of their CFRs, taking into account the local context.

## Conclusion

CFRs felt they made a valuable contribution: they wished to maintain basic skills while having the opportunity to develop these and they wanted information on their contribution to outcomes. As CFR schemes expand and their contribution to services increases, further research is needed on their effects, outcomes, and costs. A wider understanding of stakeholder perceptions of CFR and CFR schemes is also needed.
